# Efficacy of Manual Ventilation Techniques During Cardiopulmonary Resuscitation in Dogs

**DOI:** 10.3389/fvets.2018.00239

**Published:** 2018-10-01

**Authors:** Kate Hopper, Marlis L. Rezende, Angela Borchers, Steven E. Epstein

**Affiliations:** ^1^Department of Veterinary Surgical and Radiological Sciences, School of Veterinary Medicine, University of California, Davis, Davis, CA, United States; ^2^Department of Clinical Sciences, College of Veterinary Medicine and Biomedical Sciences, Colorado State University, Fort Collins, CO, United States; ^3^William R. Pritchard Veterinary Medical Teaching Hospital, School of Veterinary Medicine, University of California, Davis, Davis, CA, United States

**Keywords:** rescue breathing, bag-valve mask, cardiopulmonary resuscitation, oxygen, carbon dioxide, mouth-to-nose

## Abstract

The efficacy of ventilation of dogs during cardiopulmonary resuscitation (CPR) with a tight fitting face mask or mouth-to-nose rescue breathing has not been evaluated. Twenty-four purpose bred research dogs: Dogs were randomized to be ventilated by cuffed orotracheal tube, tight fitting face mask, mouth-to-nose breathing or compressions only during CPR (*n* = 6 in all groups). Orotracheal tube and face mask ventilation was performed on room air. Chest compressions were performed during the experimental procedure. Arterial blood gases were performed prior to euthanasia (baseline), at 3 min and at 6 min of CPR. PaO_2_ and PaCO_2_ were compared for each time point and each group. There was no difference in PaO_2_ or PaCO_2_ between groups at baseline. At 6 min all groups had a significantly higher PaCO_2_ (*P* ≤ 0.005) and the facemask and compression only groups had a significantly lower PaO_2_ (*P* < 0.02) when compared to the orotracheal tube group. There was no difference between the PaO_2_ of the mouth-to-nose group compared to the orotracheal tube group at 3 or 6 min. Gastric distension, regurgitation, gas leakage around the mouth, and ineffective breaths were all noted in both the face mask and mouth-to-nose group. The results of this study supports that orotracheal intubation is the preferred technique for ventilation during CPR in dogs. When orotracheal intubation is not possible, face mask ventilation or mouth-to-nose ventilation would be reasonable alternatives. When oxygen supplementation is available, face mask ventilation is likely to be superior. Appropriate training for both face mask and mouth-to-nose ventilation techniques is recommended.

## Introduction

Artificial ventilation during resuscitation for in-hospital cardiopulmonary arrest or respiratory arrest in dogs and cats is routinely performed by manual ventilation via a cuffed orotracheal tube. When orotracheal intubation is not feasible the veterinary CPR guidelines, known as RECOVER, state “it is reasonable to recommend mouth-to-snout rescue breathing for dogs and cats with respiratory arrest or with cardiopulmonary arrest in a 30:2 ratio with chest compressions when endotracheal intubation is not available (1). Bag-mask ventilation may be an effective option in dogs and cats but equipment designed specifically for animals is required.“ The efficacy of these alternative methods for ventilation in small animals has not been evaluated.

In apneic anesthetized human patients, manual ventilation via a face mask has been shown to provide adequate ventilation and oxygenation (2, 3). Mask ventilation during CPR of adult human patients is recommended when there are two trained rescuers present and has been associated with similar or better neurologic outcomes when compared to more advanced methods of airway management, although there is a risk of regurgitation and pulmonary aspiration (3–5). Mask ventilation has several possible limitations including leakage of air from the seal over the face, loss of gas volume into the stomach instead of the lungs and airway obstruction due to head position (6, 7). The American Heart Association guidelines for adult CPR states that “bag-mask ventilation is a challenging skill that requires considerable practice for competency” (3).

Mask ventilation may be utilized in veterinary medicine when intubation is unsuccessful or the equipment for intubation is unavailable, although descriptions of mask ventilation in animals are sparse. A study in anesthetized cats comparing two bag-mask devices reported that adequate PaCO_2_ could be maintained, although no measure of oxygenation was performed in this study (8). A report of CPR in rabbits described 7 rabbits that were ventilated by face mask and 5 of these rabbits had return of spontaneous circulation (9). The RECOVER initiative developed evidence based guidelines for small animal CPR and concluded that mask ventilation may be an effective option in dogs and cats although no strong recommendation could be made given the lack of evidence (1). Anecdotally mask ventilation appears effective in dogs and cats although concerns have been raised regarding the ability to get a tight fitting face mask for animals and the importance of keeping the neck extended to prevent airway obstruction.

Mouth-to-mouth rescue breathing has a long history in human medicine. In healthy, anesthetized, paralyzed adult humans, mouth-to-mouth ventilation can maintain normal oxygen saturation and ventilation (10, 11). Mouth-to-mouth ventilation is recommended for lay person CPR and single trained rescuer CPR in people (3). Given the very different anatomy of small animals compared to people, the efficacy of mouth-to-nose breathing cannot be extrapolated from human studies. There have been no studies evaluating mouth-to-nose ventilation in animals although it has been reported anecdotally. One case report in a dog with cervical spinal trauma and associated respiratory arrest describes the owners providing mouth-to-nose ventilation until they reached a veterinary facility (12).

The objective of this study was to assess the effectiveness of manual ventilation via face mask and mouth-to-nose rescue breathing during CPR in dogs. The specific aims were to evaluate the oxygenation and ventilation achieved by these techniques, the practicalities of providing each ventilation technique and any noted complications.

## Materials and methods

### Animals

Purpose bred research dogs that were under general anesthesia for an unrelated study for laparoscopic surgery were enrolled. At the conclusion of this procedure, the current study was commenced and both were approved by the Colorado State University institutional animal care and use committee.

At the end of the initial study the dogs remained orotracheally intubated, under general anesthesia with isoflurane in 100% oxygen and an intravenous infusion of morphine (0.1 mg/kg/h), ketamine (10 mcg/kg/min) and lidocaine (30 mcg/kg/min). Electrocardiograph, direct arterial blood pressure, body temperature, ETCO_2_ and pulse oximetry were continuously monitored. The dogs had a 20 G peripheral venous and arterial catheter (20G 48 mm BD Insyte; Becton Dickinson Infusion Therapy Systems Inc., UT, USA) in place. After complete evacuation of the abdomen, the animals were positioned in right lateral recumbency and transitioned from isoflurane in oxygen to total intravenous anesthesia with a propofol constant rate infusion (2 mg/kg loading dose, followed by 0.2–0.4 mg/kg/min) as needed to maintain a surgical plane of anesthesia. The dogs were disconnected from the anesthetic machine and attached to a bag valve resuscitator (Bag valve resuscitator, Vital Signs Inc., GE Healthcare company, NJ, USA) and manually ventilated on room air, targeting an ETCO_2_ of 30 mmHg and an SpO_2_ of 92–94%. After 30 min of manual ventilation, baseline arterial blood samples were taken using purpose made heparinized syringes (SafePico Aspirator syringes, Radiometer Medical ApS, Denmark) for blood gas analysis (800 ABL Flex; Radiometer America Inc., CA, USA). The arterial blood gas values were not temperature corrected.

At baseline, the target arterial blood gas values were a PaCO_2_ of 30–40 mmHg and a PaO_2_ of 70–75 mmHg. If after 30 min the baseline values were not within these ranges, manual ventilation was continued as described and arterial blood samples were evaluated every 15 min until the appropriate baseline values were achieved.

Euthanasia was performed via injection of pentobarbital 120 mg/kg IV. The propofol and morphine, ketamine and lidocaine infusions were stopped at this time. Death was confirmed by absence of heart sounds on thoracic auscultation and loss of a pulsatile waveform on the direct arterial blood pressure monitor. Sixty seconds following confirmation of death, basic life support was initiated.

### Cardiopulmonary resuscitation

The method of ventilation used was randomized to one of four techniques (see below). For ventilation techniques that did not require orotracheal intubation, the dogs were extubated during the 60 s following confirmation of death. External chest compressions were performed at ~100 compressions per minute with hands placed over the heart, for a period of 6 min. Chest compressions were performed in 2 min cycles and the person performing chest compressions was changed for each cycle with less than a 10 s interruption in chest compressions. No other CPR interventions were performed. Arterial blood samples for blood gas (Bag valve resuscitator, Vital Signs Inc., GE Healthcare company, NJ, USA) analysis were evaluated at 3 and 6 min following initiation of chest compressions.

At the end of the 6 min study period the dogs were evaluated including thoracic auscultation and direct arterial blood pressure waveform analysis to confirm death.

### Ventilation technique

Twenty-four dogs were randomized to one of four manual ventilation techniques; bag valve resuscitator (Bag valve resuscitator (Adult), Vital Signs Inc., GE Healthcare company, NJ, USA) with orotracheal intubation on room air, bag valve resuscitator with tight fitting face mask (Anesthesia Mask Canine, Jorgensen Labs, CO, USA) on room air, mouth-to-nose breathing or no ventilation (chest compression only) CPR (*N* = 6 for each group). The orotracheal group were ventilated at a respiratory rate of 10 breaths per minute with a tidal volume to generate an adequate chest rise and an inspiratory time of 1 s. For the tight fitting face mask and mouth-to-nose techniques, a 30:2 compression to breath ratio was used and a single investigator (KH) performed all mouth-to-nose breathing.

Any difficulties or complications experienced with any of the ventilation techniques during the study was recorded with specific evaluation of evidence of gas leakage during breaths, gastric distension, and evidence of regurgitation. The presence of gastric distension and evidence of regurgitation was based on abdominal palpation and visual evaluation of the mouth, nose and oropharynx of the dogs after end of the 6 min experimental period.

### Statistics

Data was analyzed for normality with a Shapiro–Wilk normality test and normally distributed data are reported as mean ± standard deviation. PaO_2_ and PaCO_2_ values were compared at baseline, 3 min, and 6 min between ventilation techniques with a one-way ANOVA with a *post-hoc* Tukey test corrected for multiple comparisons when appropriate. Within a ventilation technique group PaO_2_ and PaCO_2_ values were compared at the three times points with a repeated measures ANOVA with a *post-hoc* Tukey test corrected for multiple comparisons when appropriate. A *P* < 0.05 was considered significant and all testing was done with commercially available software (Prism 7.0, Graph Pad, La Jolla, CA).

## Results

A total of 26 hound type, female dogs were randomized for the study and the results from 24 dogs with a mean body weight of 27 ± 4.9 kg are reported. The experiment in two dogs had to be repeated because arterial blood samples could not be obtained in one dog in the face mask group and no ventilations were effective in one dog in the mouth-to-nose group. A summary of PaO_2_ and PaCO_2_ values is presented in Table 1.

**Table 1:** Mean and standard deviation values of PaO_2_ and PaCO_2_ values of dogs during closed chest cardiopulmonary resuscitation receiving one of four methods of ventilation (*N* = 6 for all groups).

	**PaO_2_ mmHg**	**PaCO_2_ mmHg**
**BASELINE**		
Orotracheal intubation	82 ± 17.5	40 ± 3.3
Face mask	78 ± 11.9	37 ± 1.6
Mouth-to-nose	94 ± 6.7	41 ± 4.7
No ventilation	82 ± 6.6	36 ± 6.3
**3 MIN**		
Orotracheal intubation	64 ± 14.5	24 ± 5.8
Face mask	43 ± 10.1	36 ± 5.1
Mouth-to-nose	58 ± 16.5	39 ± 5.8
No ventilation	30 ± 8.1	35 ± 5.8
**6 MIN**		
Orotracheal intubation	62 ± 16.0	24 ± 9.0
Face mask	35 ± 13.2	42 ± 7.7
Mouth-to-nose	44 ± 16.2	45 ± 7.6
No ventilation	22.3 ± 3.7	41 ± 6.5

### Oxygenation

**Figure 1:**
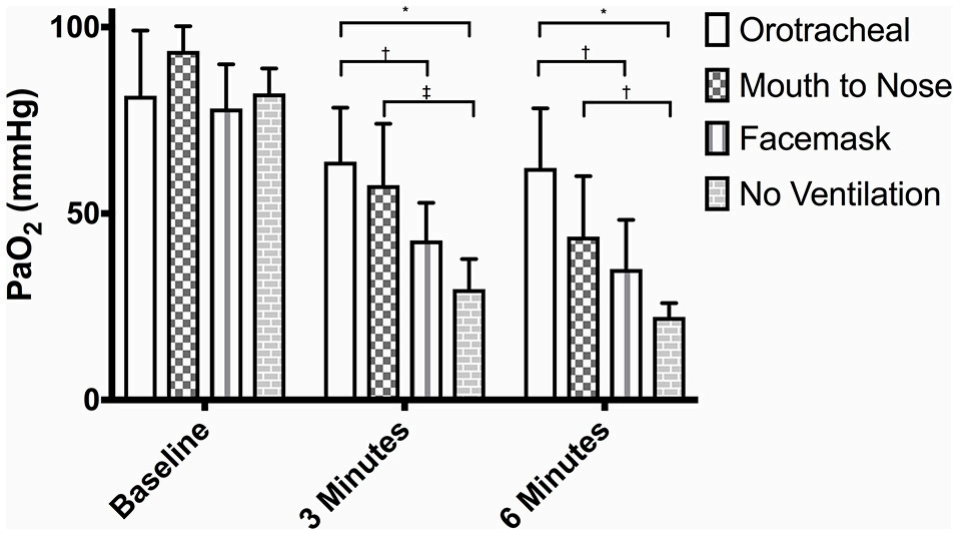
Mean PaO_2_ of dogs at baseline and at 3 and 6 min of cardiopulmonary resuscitation when ventilated via one of four techniques without supplemental oxygen. Bars represent standard deviation. *P < 0.001, ^†^*P* < 0.05, ^‡^*P* < 0.01. Orotracheal, ventilation with bag resuscitator and cuffed orotracheal tube; Mouth-to-nose, rescue breaths with mouth over the nose; face mask, ventilation with bag resuscitator and a tight fitting face mask; No ventilation, compression only CPR.

**Figure 2:**
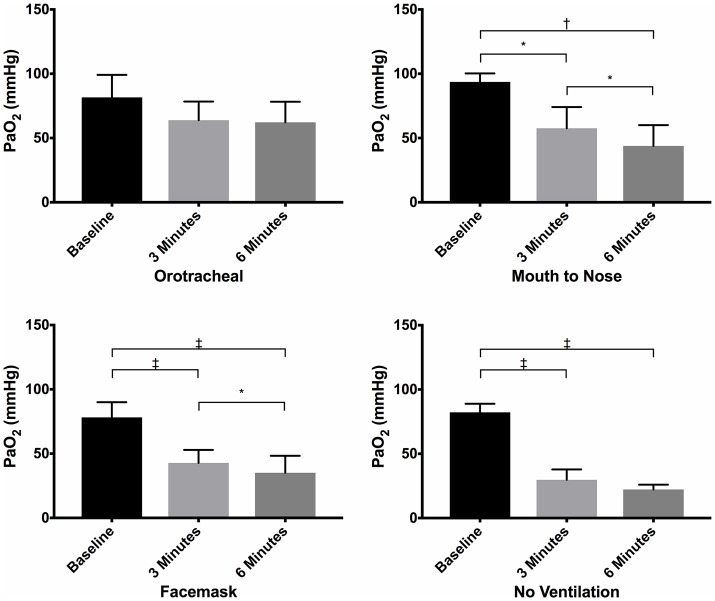
Mean PaO_2_ of dogs at baseline and at 3 and 6 min of cardiopulmonary resuscitation for each ventilation technique. Bars represent standard deviation. *P < 0.05, ^†^*P* < 0.005, ^‡^*P* < 0.0005. Orotracheal, ventilation with bag resuscitator and cuffed orotracheal tube; Mouth-to-nose, rescue breaths with mouth over the nose; face mask, ventilation with bag resuscitator and a tight fitting face mask; No ventilation, compression only CPR.

There were no differences in PaO_2_ between groups at baseline (Figure 1) and the PaO_2_ of the orotracheal group at 3 and 6 min did not differ from baseline. The PaO_2_ of the face mask, mouth-to-nose, and compression only groups was significantly lower at 3 and 6 min than at baseline (Figure 2). The PaO_2_ at 6 min was significantly lower than at 3 min in the facemask and mouth-to-nose groups (Figure 2).

At both 3 and 6 min, the PaO_2_ in the orotracheal group was significantly higher than in the face mask and compression only groups, but not the mouth-to-nose group (Figure 1). The PaO_2_ of the mouth-to-nose group had a significantly higher PaO_2_ than the compression only group at both 3 and 6 min (Figure 1).

### Ventilation

**Figure 3:**
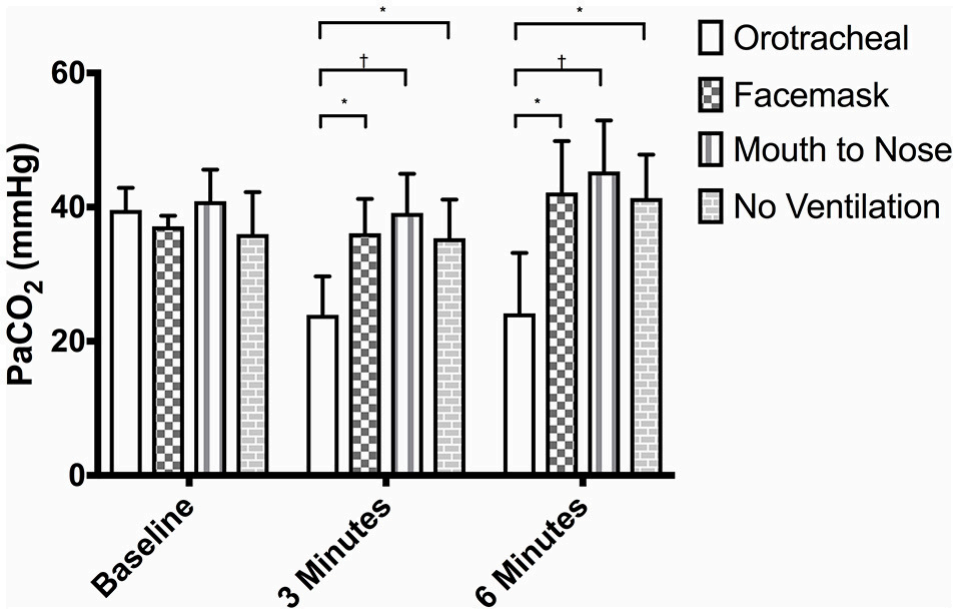
Mean PaCO_2_ of dogs at baseline and at 3 and 6 min of cardiopulmonary resuscitation when ventilated via one of four techniques without supplemental oxygen. Bars represent standard deviation. *P ≤ 0.01, ^†^*P* < 0.001. Orotracheal, ventilation with bag resuscitator and cuffed orotracheal tube; Mouth-to-nose, rescue breaths with mouth over the nose; face mask, ventilation with bag resuscitator and a tight fitting face mask; No ventilation, compression only CPR.

**Figure 4:**
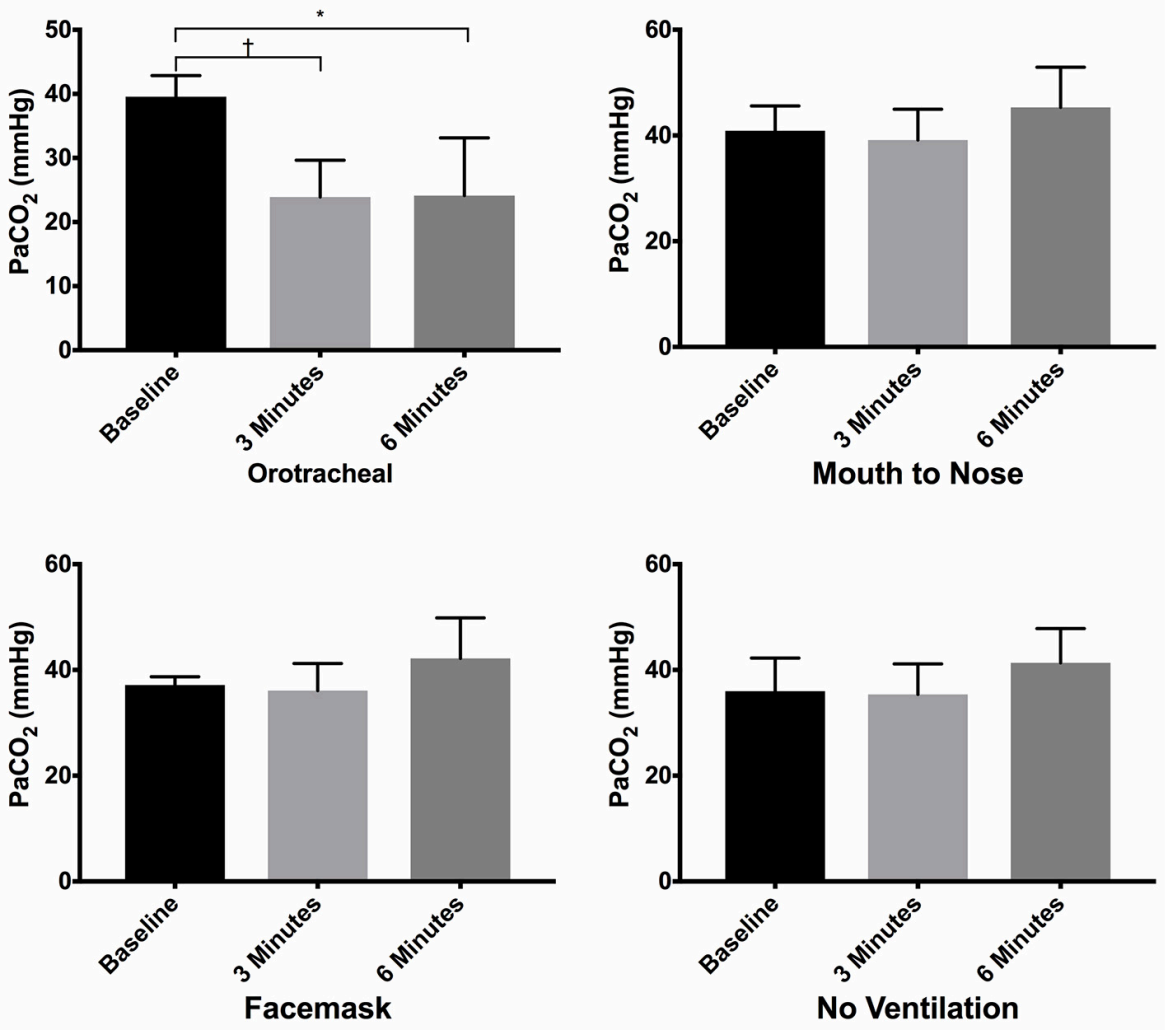
Mean PaCO_2_ of dogs at baseline and at 3 and 6 min of cardiopulmonary resuscitation for each ventilation technique. Bars represent standard deviation. *P < 0.05, ^†^*P* < 0.005. Orotracheal, ventilation with bag resuscitator and cuffed orotracheal tube; Mouth-to-nose, rescue breaths with mouth over the nose; face mask, ventilation with bag resuscitator and a tight fitting face mask; No ventilation, compression only CPR.

There was no difference in the PaCO_2_ between groups at baseline (Figure 3). The PaCO_2_ of the orotracheal group was significantly lower at 3 min and at 6 min when compared to baseline. There was no difference in the PaCO_2_ between time points for the face mask, mouth-to-nose and compression only groups (Figure 4). At both 3 and 6 min the PaCO_2_ was lower in the orotracheal group compared to all other groups. There was no difference in the PaCO_2_ between the face mask, mouth-to-nose and compression only groups at 3 or 6 min (Figure 3).

No complications were noted in the orotracheal intubation and compression only groups. In the face mask group, head extension and two hands to keep the mask tightly pressed against the mouth was necessary to generate effective chest rise with ventilation. Despite these efforts, in 3 out of 6 dogs ineffective breaths were noted. Gastric distension was evident in one of six dogs and leakage of air around the mask was noted in four of six dogs. In one dog it was noted that there was intermittent contact of the mask against both corneas during the CPR period.

In the mouth-to-nose group, head extension and occlusion of the lips was necessary to generate effective breaths. Ineffective breaths were noted in two of six dogs in the mouth-to-nose group, regurgitation during CPR was noted in two of six dogs and gastric distension was evident in three of six dogs. In addition to the issues noted in the 6 dogs in this group, mouth-to-nose breathing failed to generate any effective breaths in a 7th dog for the entire period of CPR. The PaO_2_ at 3 and 6 min for this dog was 35 and 31 mmHg, respectively and the PaCO_2_ at 3 and 6 min was 43 and 49 mmHg, respectively. There was no clear reason for the inability to ventilate this dog on oral examination after the experimental period. No dog in any group had return of spontaneous circulation during the experimental period.

## Discussion

In this study comparing ventilation techniques during CPR in dogs without supplemental oxygen, orotracheal intubation provided higher oxygenation than face mask ventilation or compression only CPR. Although the level of oxygenation achieved with face mask ventilation appeared lower than that with mouth-to-nose breathing with a larger difference at the 6 min point, it did not reach statistical significance. Ventilation with orotracheal intubation resulted in consistently lower PaCO_2_ values than other groups. Both face mask and mouth-to-nose ventilation techniques did not consistently produce effective breaths and were associated with complications.

In order to compare the effectiveness of mouth-to-nose ventilation, orotracheal and face mask ventilation in this study was performed on room air. As such, the PaO_2_ values found in this study are expected to be lower than those in a clinical setting where supplemental oxygen would be used for ventilation. In addition, this experiment was performed at an altitude of ~5,000 feet (barometric pressure ranged from 628 to 642 mmHg), which will lower PaO_2_ values compared to those obtained at sea level. A previous study in pigs ventilated via orotracheal intubation on room air had similar results to this study with a reported PaO_2_ of 69 ± 15 mmHg after 5 min of cardiopulmonary arrest (13). In comparison, experimental animal studies have reported PaO_2_ values in the range of 300–400 mmHg with orotracheal intubation when ventilated on 100% oxygen during closed chest CPR (14–16).

The PaO_2_ declined over the 6 min experimental period in this study for both the face mask and mouth-to-nose groups. A deterioration in PaO_2_ during CPR has been reported in previous experimental studies and has been attributed to atelectasis and pulmonary injury secondary to chest compressions (15). The higher PCO_2_ in the face mask and mouth-to-nose groups suggests less effective ventilation and may be consistent with atelectasis occurring in these animals. Massive pulmonary aspiration during CPR is another possible cause of deteriorating oxygentation in the non-intubated animals. In human medicine, the optimal airway management during CPR is uncertain (17). This reflects the difficulties and risks associated with orotracheal intubation in people. In contrast, orotracheal intubation is relatively simple in dogs and it is considered the optimal airway management option in CPR (1). When orotracheal intubation is not possible, due to patient anatomy, lack of equipment or operator experience, a non-invasive airway to allow ventilation during CPR or respiratory arrest is necessary, as evidenced by the no ventilation group having the lowest PaO_2_ values.

Ventilation via a face mask and a bag valve resuscitator (also known as bag-mask ventilation) is common in human CPR and is well-described in the literature, but to the author's knowledge it has not been previously evaluated in dogs. In this study, a clear plastic face mask with a rubber diaphragm was used to give a tight fitting seal around the muzzle. The oxygenation provided using this technique was not different than that of the compression only group. This may be due to the difficulties in consistently producing effective breaths. The operator had to hold the mask tightly against the face and occlude the lips to prevent air leakage. The head and neck needed to be maintained in extension, as often small changes in position resulted in an ineffective breath and a second operator to help keep the neck and head appropriately positioned was frequently needed. The oxygenation achieved with this technique in this study was poorer than expected. In a clinical setting, face mask ventilation would allow ventilation with an enriched oxygen source which may increase the PaO_2_ attained. Although, given the lack of difference in PaO_2_ between the face mask group and compression only group in this study, the impact of supplemental oxygen with face mask ventilation during CPR cannot be predicted. Gastric distension was evident in one of the dogs following face mask ventilation in this study. As gastric distension was identified on physical examination only, it is possible that it occurred more frequently than reported. A prospective human clinical study reported a much higher rate of regurgitation with bag-mask ventilation (12.4%) than with laryngeal mask airway ventilation (3.5%) during CPR (7).

Mouth-to-nose ventilation has not been evaluated in dogs previously while mouth-to-mouth ventilation in human CPR patients is well-described. Mouth-to-nose ventilation is predicted to be less effective in providing oxygenation given the limited FIO_2_. The gas delivered by mouth for rescue breaths has a higher PCO_2_ and lower PO_2_ than room air. Studies have reported the fraction of oxygen in expired gas to be in the range of 15.9–17.9% (18, 19). The fraction of expired oxygen changes with the number of ventilations provided by the rescuer. A study comparing compression:ventilation ratios found that a ratio of 30:2 provided a higher fraction of expired oxygen than 15:2, but lower than a ratio of 30:5 (19). With increased ventilation frequency, there is hyperventilation of the rescuer which causes reduced fraction of carbon dioxide in the exhaled gas. The tidal volumes generated by the rescuer is also likely to impact the fraction of expired oxygen.

Another challenge of mouth-to-nose ventilation is generating effective breaths. In this study it was necessary to hold the mouth closed and the lips of the dogs occluded to prevent air leakage. As with the face mask ventilation, keeping the head and neck extended was also important. In one dog, no effective breaths could be generated for the entire 6 min period. There were no anatomical abnormalities evident on evaluation of the oropharynx or larynx of this dog at the end of the experimental period and obstruction of the nasal passage was suspected. Overall, in the opinion of the investigators of this study, it was easier to produce consistent breaths using the mouth-to-nose technique than the face mask technique. The dogs in this study were dolichocephalic, mouth-to-nose ventilation of dogs of other anatomical type maybe less effective. Gastric distension and regurgitation was common in the mouth-to-nose ventilation group. In out of hospital, human CPR patients mouth-to-mouth ventilation was associated with a significantly increased risk of regurgitation compared to compression only CPR, or no CPR (20). These findings further support the emphasis on orotracheal intubation for CPR in small animal patients, where it is usually possible with minimal complications.

Compression only CPR was included in this study for comparison purposes and it was associated with a very low PaO_2_. This value was similar to values reported in pigs after 5–15 min of compression only CPR (21). Compression only CPR maybe associated with poorer outcomes than conventional CPR in human clinical patients (22–24). Despite this, it is currently recommended for out of hospital CPR in humans for untrained rescuers, as it has been found to be associated with a higher rate of bystander CPR provision (25, 26).

The PaCO_2_ is determined by CO_2_ production and alveolar minute ventilation (27). In this study, the animals ventilated via orotracheal intubation developed hypocapnia during CPR, despite a similar degree of ventilation as provided at baseline. This likely reflects the decrease in CO_2_ production and reduced blood flow associated with cardiopulmonary arrest and has been reported in previous experimental animal CPR studies (21, 28–30). As ventilation was performed manually in this study, we cannot guarantee what tidal volume was provided during the experiment. We used a respiratory rate of 10 breaths per minute as recommended by the current veterinary guidelines (1). The optimal tidal volume during CPR in dogs is not currently known but our results suggest a lower tidal volume or respiratory rate would have been ideal in our orotracheal group. The PaCO_2_ in the face mask and mouth-to-nose ventilation groups was not different than the compression only CPR group. This may in part be related to inherent limitations of these techniques. Face mask ventilation increases dead space, necessitating a higher minute ventilation to maintain PaCO_2_, while mouth-to-nose ventilation has higher inspired PCO_2_ (18, 19, 31, 32). In addition, using a 30:2 compression:ventilation ratio will provide less total ventilation per minute than breathing at 10 breaths per minute as was performed in the orotracheal group. In both the face mask and mouth-to-nose group, not all breaths were considered effective which would further compromise the total alveolar ventilation provided. The optimal PaCO_2_ during CPR is currently unknown and generally a normal PaCO_2_ is targeted (21). Hypocapnia during CPR has been associated with a higher mortality, likely due to impaired venous return with hyperventilation (16, 33). The levels of PaCO_2_ attained with face mask and mouth-to-nose ventilation in this study were generally higher than considered normal for dogs and suggests inadequate alveolar minute ventilation was acheieved with these techniques.

This study has several limitations. The sample size was small which limits the ability to find significance between groups. The dogs used for this study had been anesthetized for several hours for laparoscopic procedures prior to the experimental period. As a result, these dogs may have developed pulmonary or cardiovascular abnormalities that may have impacted our results. Although this was less than ideal, sacrifice of healthy animals for this study alone was not deemed justified. The chest compressions in this study were performed manually with multiple compressors so variability in chest compression quality and rate is inevitable. In addition, as only basic life support was provided, maximal cardiac output may not have been achieved. Changes in blood flow does have some impact on arterial blood gas values and the potential variability in cardiac output during this experiment may have contributed to some variability in the results, ultimately reducing the likelihood of finding significant differences between groups.

When comparing ventilation techniques, it has been suggested that three main criteria should be considered: (1) ease of use; (2) efficacy in maintaining oxygenation and ventilation; (3) frequency of complications (34). Using these criteria, the results of this study supports that orotracheal intubation is the preferred technique for ventilation during CPR in dogs. When orotracheal intubation is not possible, the results of this study would suggest that either face mask ventilation or mouth-to-nose ventilation would be reasonable alternatives. When oxygen supplementation is available, face mask ventilation is likely to be superior. Appropriate training for both face mask and mouth-to-nose ventilation techniques is recommended.

## Author contributions

KH, MR, and AB designed the study and performed the experimental procedures. SE analyzed the data and KH wrote the manuscript. All authors contributed to read and approved the manuscript.

### Conflict of interest statement

The authors declare that the research was conducted in the absence of any commercial or financial relationships that could be construed as a potential conflict of interest. The reviewer LG and the handling Editor declared their shared affiliation
